# Illustration of a patient with stroke developing ipsilateral tremor-like grasp phenomenon

**DOI:** 10.1055/s-0043-1763488

**Published:** 2023-03-22

**Authors:** Halil Onder, Asim Kilic, Selcuk Comoglu

**Affiliations:** 1 Diskapi Yildirim Beyazit Training and Research Hospital, Neurology Clinic, Ankara, Turkey.

**Figure 1:**
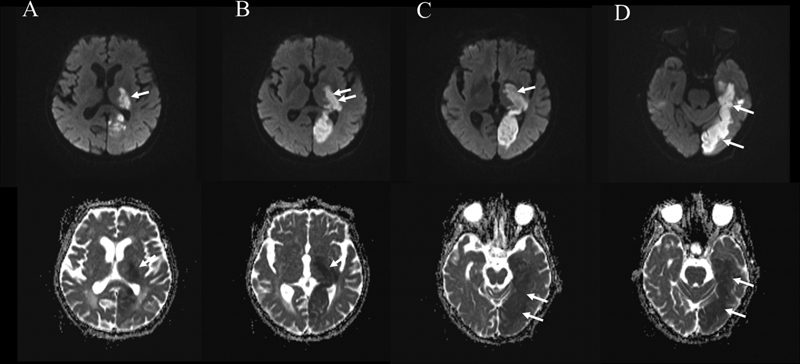
The diffusion-weighted imaging (DWI) and apparent diffusion coefficient (ADC) sequences show the acute diffusion restriction in the vascular territory of the right posterior cerebral artery. The figure shows the lateral portion of the thalamus ( **A-C** ), the posterior limb of the internal capsule ( **B-C** ), the inferior temporal lobe ( **D** ), and the occipital lobe ( **C-D** ) (arrows). The DWI sequences are illustrated in the upper part of the figure, and the ADC images are illustrated in the lower part of the figure.

An 83-year-old right-handed woman with coronary artery disease (CAD) was admitted due to acute-onset confusion, visual impairment, and right-sided paresis. Upon admission, the patient was lethargic and disorientated in terms of time and place. The visual field examination revealed right-sided homonymous hemianopsia, and the motor examination revealed paralysis on the right side (which was of grade 4 according to the medical research council). She also had the grasp reflex on her left hand. A Cranial magnetic resonance imaging (MRI) scan revealed acute infarction in the territory of the left posterior cerebral artery (PCA) ( Figure 1 ). On the second day of follow-up, the patient developed ipsilateral involuntary movements of her left hand and foot that were compatible with the tremor-like grasp phenomenon ( Video 1 ). 1 2 Of note, a routine electroencephalogram (EEG) did not show an epileptiform discharge. The movements were fully recovered one week later without additional interventions. We herein report a very rare entity of a motor symptom ipsilateral to an acute brain injury that has been hypothesized to be due to hyperexcitation of the frontal lobe, contralateral to the movements. 2

**Video 1** Video of the patient recorded on the second day of admission, showing involuntary movements in her left hand and foot that were stereotypical, sometimes rhythmical, and accompanied by groping or picking-like movements. Of note, these tremor-like involuntary movements were ipsilateral to the side of the infarction. https://www.arquivosdeneuropsiquiatria.org/anp-2022-0160-video/

